# What is the Cost of Selectivity? Selective and Nonselective Alpha Blockade Costs Associated with Adrenalectomy for Pheochromocytoma

**DOI:** 10.1245/s10434-026-19255-3

**Published:** 2026-02-20

**Authors:** Lauren R. Kelz, Jesse E. Passman, Colleen Brensinger, Lily Owei, Sara P. Ginzberg, Heather Wachtel

**Affiliations:** 1 Department of Surgery, Hospital of the University of Pennsylvania, Philadelphia, PA USA; 2 The Center for Clinical Epidemiology and Biostatistics, Perelman School of Medicine of the University of Pennsylvania, Philadelphia, PA USA

**Keywords:** Alpha blockade, Pheochromocytoma, Cost-effectiveness, Adrenalectomy, Phenoxybenzamine, Doxazosin, Prazosin

## Abstract

**Background:**

Alpha-blockade is utilized in the preoperative preparation of patients with pheochromocytomas. Both selective and nonselective alpha-blockade are safe with equivalent clinical outcomes; the goal of this study was to evaluate the comparative costs of selective and nonselective alpha-blockade.

**Patients and Methods:**

We performed a retrospective cohort study (2004–2022) of patients who underwent adrenalectomy for pheochromocytoma with preoperative alpha-blockade from Optum’s deidentified Clinformatics® Data Mart Database. Patients were stratified by treatment with selective (prazosin, doxazosin, terazosin) or nonselective (phenoxybenzamine) alpha-blockade. Primary outcomes were: (1) costs of alpha-blockade in the 30 days prior to surgery (AB) and (2) adjusted standard costs from admission to discharge (AC). Secondary outcomes included length of hospital stay (LOS), intensive care unit (ICU) admission, costs 30 days after discharge (AD), and cumulative costs (CC).

**Results:**

In total, 384 patients received selective, and 418 patients received nonselective alpha-blockade. The median age was 58 years (IQR 21 years). Median AB was significantly lower in the selective compared with nonselective alpha-blockade group ($19.73 versus $1033.70, *p* < 0.001). Median AC ($31,104.47 versus $31,471.90, *p* = 0.428) and AD ($790.78 versus $715.10, *p* = 0.074) were not significantly different between selective compared with nonselective alpha-blockade groups. On multivariable regression modeling, higher Elixhauser score (coeff.: $1,801.20, *p* < 0.001) and longer LOS (coeff.: $1842.33, *p* < 0.001) were associated with higher CC, while age (coeff.: $− 143.22, *p* = 0.044) was associated with lower CC. Notably, alpha-blockade strategy was not significantly associated with CC.

**Conclusions:**

When compared with nonselective alpha blockers, selective alpha blockers are associated with lower medication costs but equivalent hospitalization and post-hospitalization costs.

**Supplementary Information:**

The online version contains supplementary material available at 10.1245/s10434-026-19255-3.

Pheochromocytomas are neuroendocrine tumors derived from the chromaffin cells of the adrenal medulla. The majority produce catecholamines, leading to hemodynamic signs and symptoms such as hypertension, palpitations, or arrhythmias.^1,2^ Surgical resection is the first line of treatment for isolated tumors, with minimally invasive adrenalectomy preferred.^3^ Preoperative preparation with alpha-blockade is standard of care to mitigate the release of catecholamines upon resection and minimize cardiovascular morbidity and mortality.^4–6^

Two primary categories of alpha-blockers are used in the preoperative management of pheochromocytoma—selective (terazosin, doxazosin, and prazosin) and nonselective (phenoxybenzamine). Clinical outcomes are comparable and there is no clear advantage to selective versus nonselective alpha-blockade, although minor differences exist.^1^ One contemporary meta-analysis showed that nonselective alpha-blockade is associated with lower intraoperative maximum blood pressure and less frequent use of vasodilators at the time of resection.^7^ Conversely, selective blockade may be associated with a greater likelihood of transient hypotension during surgery and greater need for vasopressor support postoperatively.^8^ Despite the clinical equipoise, selective alpha-blockers are less expensive and are more widely accessible, which has led to a shift away from nonselective alpha-blockade in recent years.^9^ Further, selective alpha-blockade may be associated with fewer side effects and may have better patient compliance due to options for less frequent dosing regimens.^7^

In the absence of a clear clinical advantage for either selective or nonselective blockade, nonclinical factors may play a more prominent role in management strategies. Despite the lower medication costs associated with selective alpha-blockade, there is a dearth of data on the overall costs across the trajectory of perioperative care for pheochromocytoma. Thus, our study aims to evaluate the comparative costs associated with selective and nonselective alpha-blockade at the time of adrenalectomy for pheochromocytoma.

## Patients and Methods

### Data Source

Optum’s deidentified Clinformatics® Data Mart Database (Optum Clinformatics®) is derived from a database of administrative health claims for members of large commercial and Medicare Advantage health plans. Optum Clinformatics® utilizes medical and pharmacy claims to derive patient level enrollment information, healthcare costs, and resource utilization information. The population is geographically diverse, spanning all 50 states and is statistically deidentified under the Health Insurance Portability and Accountability Act (HIPAA) Privacy Rule’s Expert Determination method and managed according to Optum® customer data use agreements. Optum Clinformatics® administrative claims submitted for payment by providers and pharmacies are verified, adjudicated, and deidentified prior to inclusion.

### Study Cohort

**Fig. 1:**
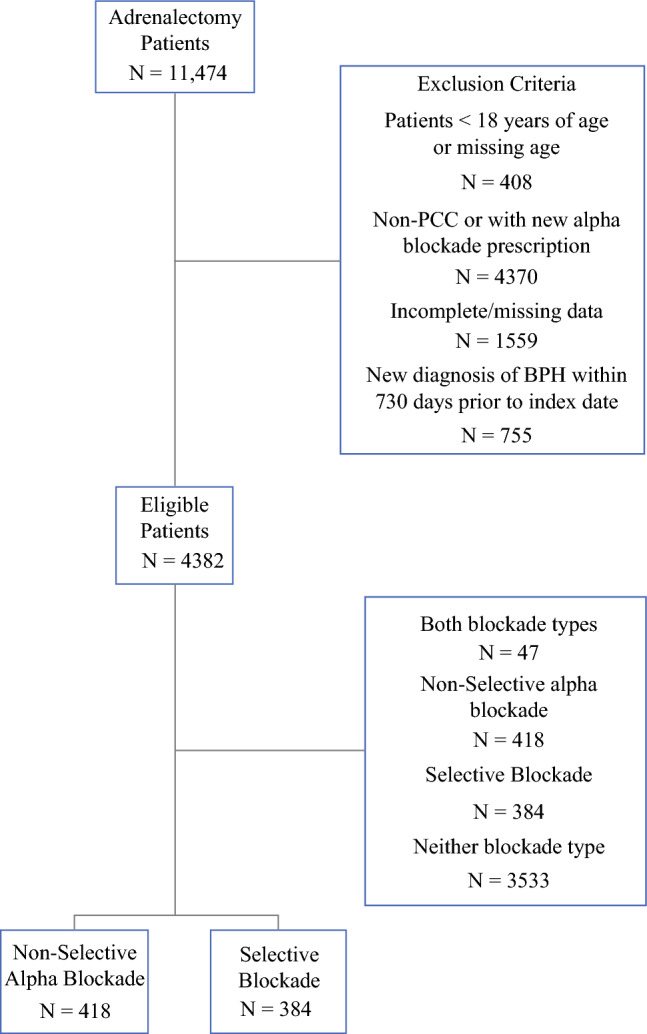
Flow chart of patient evaluation; a total of 11,474 patients were assessed for inclusion; of 4382 patients eligible for inclusion, 418 had nonselective alpha-blockade, 384 had selective alpha-blockade, 47 had both selective and nonselective alpha-blockade, and 3533 had no alpha-blockade

The initial cohort that was screened for inclusion consisted of 11,474 patients who underwent adrenalectomy between 2004 and 2022, as identified by Current Procedural Terminology (CPT) code (60540, 60545, 60650), for a diagnosis of pheochromocytoma (ICD9 1940, 2270, 2372, 2556; ICD10 C7410, C7411, C7412, C7490, C7491, C7492, D3500, D3501, D3502, E275) made ≤ 2 years of surgery, or a new prescription for alpha-blockade within 365 days of surgery and no prior alpha-blockade prescription 365–730 days prior to the date of surgery. Patients were excluded if they did not have a new alpha-blockade prescription or an adrenalectomy for a non-pheochromocytoma diagnosis (by CPT codes and ICD9/10 diagnostic codes). A total of 408 patients < 18 years of age or missing age data were excluded. Patients with incomplete/missing data or a new diagnosis of benign prostatic hypertrophy (BPH) ≤ 730 days prior to the index date were also excluded. The final cohort included 4382 patients (Fig. 1). This study was reviewed by the Institutional Review Board (IRB) of the University of Pennsylvania and deemed exempt.

### Variables

Patients were stratified by treatment as selective alpha-blockade—prazosin, doxazosin, or terazosin—or nonselective alpha-blockade—phenoxybenzamine. Patients who had been prescribed both treatments were not included in the final analysis. Other variables included age, sex, race, Elixhauser score, insurance status, household income, surgery type, length of stay (LOS), and intensive care unit (ICU) admission. Elixhauser score was derived from the total summary score, which is the sum of the 31 different comorbidity indicators. Race was categorized as white, Black, Asian, Hispanic, or unknown in accordance with the Optum Clinformatics® categories. Household income was stratified as < $40,000, $40,000–49,000, $50,000–59,000, $60,000–69,000, $60,000–74,000, $74,000–99,000, ≥ $100,000, and unknown. Insurance status was defined as commercial/private or Medicare. Adrenalectomy approach was categorized as minimally invasive or open on the basis of ICD9/10 diagnostic codes. Robotic adrenalectomy was included in the minimally invasive category (Supplement 1).

### Outcomes of Interest

**Fig. 2:**
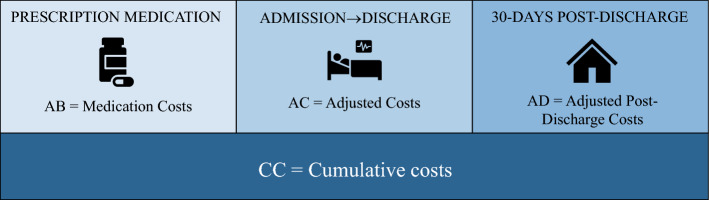
Description of cost outcomes; costs of alpha-blockade were derived from prescription medication claims (AB); adjusted standard costs were defined by all medical claims from the date of admission to the date of discharge (AC). Adjusted medical costs in the 30 days after discharge included both medical claims and prescription medication claims (AD); cumulative costs (CC) were defined as the total of adjusted standard costs, costs of alpha-blockade, and adjusted medical costs in the 30 days after surgery (CC = AB + AC + AD)

The primary outcomes were: (1) costs of alpha-blockade in the 30 days prior to surgery; and (2) adjusted standard costs from admission to discharge. Secondary outcomes included LOS, ICU admission, adjusted medical costs in the 30 days after discharge, and cumulative costs. Costs of alpha-blockade were derived from prescription medication claims (AB). AB claims are defined by Optum Clinformatics® as the allowed payment for all provider services. Adjusted standard costs were defined by all medical claims from the date of admission to the date of discharge (AC). Adjusted medical costs in the 30 days after discharge included both medical claims and prescription medication claims (AD). AC and AD claims were defined by Optum Clinformatics® as the allowed amount for facility charges. Surgeon’s fees were not included in these costs. Cumulative costs (CC) were defined as the total of adjusted standard costs, costs of alpha-blockade, and adjusted medical costs in the 30 days after surgery (CC = AB + AC + AD) (Fig. 2). Costs were adjusted by year and type of service.

### Statistical Analysis

Categorical variables were presented using frequency and percentile. Continuous variables were expressed as mean and standard deviation (SD) for normally distributed variables and as median and interquartile range (IQR) for nonnormally distributed variables. Descriptive statistics were performed. Group comparisons were performed for primary and secondary outcomes stratified by alpha-blockade type using *χ*^2^ test or Wilcoxon rank sum test, as appropriate. Univariable and multivariable linear regression of adjusted total costs were performed with covariates including age, sex, race, Elixhauser score, insurance status, household income, surgery type, LOS, and blockade type. A *p*-value of < 0.05 was considered statistically significant. Wilcoxon–Mann–Whitney test was used to estimate sample size and determine power between selective and nonselective alpha-blockade groups, using alpha 0.05 and power 0.80. Analysis was conducted with SAS®, version 9.4.

## Results

### Cohort Characteristics

Of the 4382 patients in the final study cohort, 418 received nonselective alpha-blockade and 384 patients received selective alpha-blockade (Table 1). In total, 65.7% were female and the majority (74.5%) were white. The median age was 58 years (IQR 21 years). The median Elixhauser comorbidity score was 3 (IQR 4). Most patients used private insurance (63.8%). Overall, 24.1% of patients had a household income of < $40,000 while 26.8% had a household income of ≥ $100,000. Most patients underwent a minimally invasive adrenalectomy (67.5%). The selective alpha-blockade group was significantly older (61 versus 53 years, *p* < 0.001), had a greater majority of female patients (66.9% versus 59.6%, *p* = 0.031), had lower rates of private insurance (53.7% versus 81.3%, *p* < 0.001), had a smaller percentage of household income ≥ $100,000 (24.0% versus 31.1%), and was more likely to undergo a minimally invasive adrenalectomy approach (71.1% versus 62%, *p* < 0.001).

**Table 1:** Cohort characteristics of 4382 patients with pheochromocytoma undergoing adrenalectomy with selective or nonselective alpha blockade

Characteristic	Total cohort (*n* = 4382)	Selective alpha blockade (*n* = 384)	Nonselective alpha blockade (*n* = 418)	*p*-Value
Median age, years (IQR)	58 (47, 68)	61 (50, 70)	53 (44, 62)	< 0.001
Sex				0.031
Female	65.7%	66.9%	59.6%	
Male	34.3%	33.1%	40.4%	
Race				0.223
White	74.5%	73.4%	76.9%	
Black	13.%	15.6%	10.9%	
Asian	3.1%	4.8%	4.2%	
Hispanic	9.0%	6.2%	8.1%	
Median Elixhauser score (IQR)	3 (1, 5)	3 (2, 5)	3 (1, 4)	0.002
Insurance status				< 0.001
Private	63.8%	53.7%	81.3%	
Medicare	36.2%	46.4%	18.7%	
Household income				0.002
Unknown	11.8%	15.9%	19.6%	
< $40,000	24.1%	26.0%	14.6%	
$40,000–49,000	7.0%	5.2%	6.0%	
$50,000–59,000	7.1%	7.0%	6.5%	
$60,000–74,000	10.0%	10.2%	8.1%	
$75,000–99,000	13.2%	11.7%	14.1%	
≥ $100,000	26.8%	24.0%	31.1%	
Adrenalectomy approach				< 0.001
Open	22.9%	21.9%	19.1%	
Minimally invasive	67.5%	71.1%	62.0%	
Unknown	9.6%	7.0%	18.9%	

### Perioperative Costs and Outcomes

The median cost of alpha-blockade in the 30 days prior to surgery was $313.24 (IQR $1231.79), as presented in Table 2. Median costs of alpha-blockade ($19.73 versus $1033.70, *p* < 0.001) were significantly lower in the selective compared with the nonselective alpha-blockade group. The median adjusted standard cost from surgery to discharge was $30,442.31 (IQR $23,007.17), which was not significantly different between groups (selective: $31,104.47 versus nonselective: $31,471.90). Rates of ICU admission (50.6% versus 59.8%, *p* = 0.015) were significantly lower in the selective compared with the nonselective alpha-blockade group. Median LOS (selective: 2 days versus nonselective: 3 days, *p* = 0.296), costs in the 30 days after discharge (selective: $790.78 versus nonselective: $715.10, *p* = 0.074), and cumulative costs (selective: $33,462.50 versus nonselective: $33,004.96, *p* = 0.160) were not significantly different between groups. A sensitivity analysis was performed to assess what difference in perioperative medication costs would be associated with a statistically significant difference in cumulative costs. If the entire delta in cost was due to perioperative alpha blockade, an $8436 difference in perioperative medication between groups (selective: $46,450 versus nonselective: $38,014) would have an 80% power to detect a statistically significant difference in median cumulative costs, assuming alpha 0.05.

**Table 2:** Costs and perioperative outcomes for patients with pheochromocytoma undergoing adrenalectomy, by alpha blockade type

	Selective alpha blockade (*n* = 384)	Nonselective alpha blockade (*n* = 418)	*p*-Value
*Median costs, $ (IQR)*			
Alpha blockade 30 days prior to surgery (AB)	19.73 (10.59, 40.14)	1033.70 (626.49, 3132.43)	< 0.001
Adjusted standard costs from surgery to discharge (AC)	31,104.47 (25,431.32, 45,940.47)	31,471.90 (25,718.91, 42,876.41)	0.428
Costs 30 days post-discharge (AD)	790.78 (291.59, 3509.78)	715.10 (239.00, 2031.47)	0.074
Cumulative costs (CC)	33,462.50 (27,711.61, 47,881.14)	33,004.96 (26,788.33, 45,722.40)	0.160
Median length of stay, days (IQR)	2 (1, 4)	3 (1, 5)	0.296
ICU admission	50.6%	59.8%	0.015

### Linear Regression for Adjusted Standard Costs from Surgery to Discharge (AC)

To determine the association between AC and clinical covariates, we performed linear regression modeling. On univariable linear regression, Hispanic race (coeff.: $21,620.77, *p* < 0.001), median Elixhauser score (coeff.: $2,479.48, *p* < 0.001), open surgical approach (coeff.: $14,093.72, *p* < 0.001), LOS (coeff.: $2625.10, *p* < 0.001) and ICU admission (coeff.: $5253.70, *p* < 0.001) were significantly associated with increased adjusted AC. On final multivariable regression modeling, median Elixhauser score (coeff.: $997.79, *p* < 0.001) and LOS (coeff.: $1504.15, *p* < 0.001) remained significantly associated with higher AC, as presented in Table 3. Younger age (coeff.: $− 114.77, *p* = 0.040) was significantly associated with lower AC. Alpha-blockade strategy (selective versus nonselective) was not significantly associated with AC on either univariable or multivariable modeling.

**Table 3:** Univariable and multivariable linear regression to evaluate the association between clinical characteristics and adjusted standard costs from surgery to discharge

	Univariable			Multivariable		
	Coefficient	95% CI	*p*-Value	Coefficient	95% CI	*p*-Value
Age, years	99.32	[− 86.99, 285.63]	0.296	− 114.77	[− 224.39, − 5.15]	0.040
Sex			0.907			0.296
Female	–	–		–	–	
Male	330.51	[− 5220.05, 5881.07]		1334.58	[− 1171.61, 3840.78]	
Race						
White	–	–		–	–	
Black	1327.09	[− 6947.71, 9601.89]	0.753	− 1217.71	[− 5029.63, 2594.21]	0.531
Asian	− 6659.89	[− 20,132.48, 6812.71]	0.332	− 4613.06	[− 10,893.59, 1667.48]	0.150
Hispanic/Latino	21,620.77	[10,810.58, 32,430.97]	< 0.001	2258.18	[− 2396.97, 6913.32]	0.341
Unknown	− 754.99	[− 10,681.01, 9171.03]	0.881	1135.37	[− 3918.30, 6189.04]	0.659
Elixhauser score	2479.48	[1526.05, 3432.91]	< 0.001	997.79	[510.59, 1484.98]	< 0.001
Insurance status			0.0348			0.642
Private	–	–		–	–	
Medicare	2918.97	[441.45, 11,900.94]		838.30	[− 2701.15, 4377.76]	
Household income						
Unknown	− 373.29	[− 8509.01, 7762.44]	0.928	− 3998.14	[− 7995.13, − 1.14]	0.050
< $40,000	4001.24	[− 629.67, 1127.17]	0.071	222.18	[− 3565.17, 4009.53]	0.908
$40,000–49,000	1854.65	[− 10,549.50, 14,258.81]	0.769	− 625.74	[− 6056.25, 4804.77]	0.821
$50,000–59,000	− 1642.44	[− 13,155.10, 9870.22]	0.780	− 1897.27	[− 7169.18, 3374.63]	0.480
$60,000–74,000	1039.87	[− 9167.00, 11306.75]	0.838	− 187.38	[− 4893.55, 4518.78]	0.938
$75,000–99,000	2261.25	[-6754.67, 11,277.17]	0.623	− 661.50	[− 4703.95, 3380.94]	0.748
≥ $100,000	–	–		–	–	
Adrenalectomy approach						
Unknown	5960.54	[2025.94, 13,947.02]	0.144	− 562.43	[− 4252.13, 3127.26]	0.765
Open	14,093.72	[7387.46, 20799.99]	< 0.001	2296.47	[− 817.17, 5410.11]	0.148
Minimally invasive	–	–		–	–	
Length of stay, days	2625.10	[2442.21, 2807.99]	< 0.001	1504.15	[1144.38, 1863.92]	< 0.001
Alpha blockade			0.774			
Nonselective	785.34	[− 4576.15, 6146.82]		− 1404.93	[− 3972.16, 1162.31]	0.283
Selective	–	–		–	–	
ICU admission	5253.70	[2692.39, 7815.01]	< 0.001	1726.26	[− 769.70, 4222.22]	0.175

### Linear Regression for Cumulative Costs (CC)

To further evaluate the association between cost and clinical covariates, we performed univariable and multivariable linear regression modeling for CC. On univariable regression, Hispanic race (coeff.: $21,448.72, *p* < 0.001), median Elixhauser score (coeff.: $3319.39, *p* < 0.001), open surgical approach (coeff.: $14,539.60, *p* < 0.001), LOS (coeff.: $2003.99, *p* < 0.001), and ICU admission (coeff.: $6271.71, *p* < 0.001) were significantly associated with increased CC. On final multivariable regression modeling, median Elixhauser score (Coeff: $1801.20, *p* < 0.001), and LOS (coeff.: $1842.33, *p* < 0.001) remained significantly associated with higher CC, as presented in Table 4. Median age (coeff.: $− 143.22, *p* = 0.044) was significantly associated with lower CC. Notably, alpha-blockade strategy (selective versus nonselective) was not significantly associated with CC on either univariable or multivariable modeling.

**Table 4:** Univariable and multivariable linear regression to evaluate the association between clinical characteristics and cumulative costs

	Univariable			Multivariable		
	Coefficient	95% CI	*p*-Value	Coefficient	95% CI	*p*-Value
Age, years	149.28	[− 51.74, 350.30]	0.145	− 143.22	[− 282.54, − 3.89]	0.044
Sex			0.998			0.184
Female	–	–		--	–	
Male	7.88	[− 5984.77, 6000.53]		2159.99	[− 1025.17, 5345.15]	
Race						
White	–	–		–	–	
Black	925.72	[− 8017.58, 9869.01]	0.839	− 3245.19	[− 8089.81, 1599.44]	0.189
Asian	− 9458.10	[− 24,019.11, 5102.90]	0.203	− 6776.65	[− 14,758.67, 1205.37]	0.096
Hispanic/Latino	21,448.72	[9765.21, 33,132.23]	< 0.001	2230.47	[− 3685.83, 8146.77]	0.459
Unknown	− 465.67	[− 11,193.58, 10,262.23]	0.932	-310.30	[− 6733.09, 6112.48]	0.925
Elixhauser score	3319.39	[2299.08, 4339.70]	< 0.001	1801.20	[1182.01, 2420.39]	< 0.001
Insurance status			0.008			0.471
Private	–	–		–	–	
Medicare	8399.97	[2224.11, 14,575.84]		1653.30	[− 2845.05, 6151.65]	
Household income						
Unknown	1012.66	[− 7764.36, 9789.69]	0.921	− 2847.22	[− 7927.05, 2232.62]	0.272
< $40,000	9179.42	[706.06, 17,652.77]	0.034	958.90	[− 3854.50, 5772.30]	0.696
$40,000–49,000	4167.78	[− 9214.13, 17,549.70]	0.541	1257.12	[− 5644.60, 8158.83]	0.721
$50,000–59,000	− 1660.63	− 14,080.78, 10759.51]	0.793	− 2290.90	[− 8991.06, 4409.24]	0.502
$60,000–74,000	4148.60	[− 6895.19, 15,192.40]	0.461	2226.24	[− 3754.89, 8207.37]	0.465
$75,000–99,000	2455.42	[− 7271.18, 12,182.02]	0.620	− 1008.56	[− 6146.16, 4129.05]	0.700
≥ $100,000	–	–		–	–	
Adrenalectomy approach						
Unknown	6394.24	[− 2236.06, 15,024.54]	0.146	− 988.93	[− 5678.22, 3700.35]	0.679
Open	14,539.60	[7292.72, 21,786.48]	< 0.001	622.45	[− 3334.72, 4579.62]	0.758
Minimally invasive	–	–				
Length of stay, days	2003.99	[1245.06, 2762.92]	< 0.001	1842.33	[1385.08, 2299.57]	< 0.001
Alpha blockade			0.802			0.150
Nonselective	− 741.38	[− 6529.93, 5047.16]		− 2397.01	[− 5659.75, 865.73]	
Selective	–	–		–	–	
ICU admission	6271.71	[2980.05, 9563.36]	< 0.001	1670.87	[− 1501.28, 4843.03]	0.301

## Discussion

Alpha-blockade is an essential component of perioperative management of pheochromocytoma.^4,6^ While historical use has been dominated by nonselective alpha-blockade, contemporary studies suggest clinical equipoise between selective and nonselective alpha-blockade.^1^ One of the major barriers to use of nonselective alpha-blockade has been cost, as the median costs of nonselective alpha-blockade are almost 90 times as high as selective alpha-blockade, which frequently represents an out-of-pocket expense for patients.^10^ Whether differences in preoperative blockade lead to divergent perioperative clinical care and downstream cost consequences needs remains incompletely studied. Competing alpha-blockade strategy in clinical settings presented a natural experiment and an opportunity to better understand perioperative costs associated with alpha-blockade type. In this study of the national Optum Clinformatics® Data Mart Database, we found that selective alpha-blockade was associated with lower medication costs compared with nonselective alpha-blockade. However, on multivariable regression modeling, selective alpha-blockade was not associated with any increase in hospitalization or post-hospitalization costs, suggesting that there are no downstream cost consequences associated with selective alpha-blockade.

There is a robust literature to support that selective and nonselective alpha-blockade have equivalent intraoperative and postoperative outcomes, despite slight differences in hemodynamic control and vasoactive medication requirements.^1,7,8,11–15^ One single-institution study observed no significant differences in length of hospital stay nor complication rates between alpha-blockade strategies.^8^ A similar study showed no difference in intraoperative control of hypertension, operative and postoperative blood pressure, or plasma volume control with respect to selective or nonselective alpha-blockade medication use.^16^ Aligned with previous studies, in our cohort we found that on final multivariable regression modeling, alpha-blockade type was not associated with either length of hospital stay or ICU admissions.

Despite the substantial literature on clinical outcomes, there is limited information on overall cost-effectiveness of selective versus nonselective alpha-blockade. The clinical relevance has become increasingly important as clinical practice has shifted away from nonselective alpha-blockers in recent years, which may be in part due to provider awareness of medication pricing.^9,17^ A prior study showed that the highest daily cost of phenoxybenzamine was $440.20 versus $5.06 per day for doxazosin.^10^ In addition to the stark difference in price between phenoxybenzamine and selective alpha-blockade, the cost of phenoxybenzamine has also continued to increase, rising from $722 to $9616 between 2008 and 2019 in one study of the national IBM MarketScan Database.^18^ In the MarketScan data, there was no difference in length of stay or total perioperative costs between selective and nonselective alpha-blockade. Building on the prior investigations which focused on the pre- and perioperative phases of care, our study analyzed cost longitudinally in pre-, peri-, and postoperative contexts out to 30 days after surgery. We saw no significant difference in median adjusted standard costs from surgery to discharge, median costs in the 30 days after discharge, nor median cumulative costs. Significantly, we found the median costs of nonselective alpha-blockade to be $1033.70 compared with $19.73 for selective alpha-blockade. By assessing costs across the care continuum, our findings present new information in the overall picture of treatment costs for pheochromocytomas.

This study has several limitations. The retrospective nature of the study limits our results to associative and not causal findings. In our analysis, we could not control for any selection bias and all clinical decisions were made a priori. Therefore, the choice of alpha-blockade may have been decided by other factors not completely captured in this database. One significant limitation was the lack of biochemical data; in practice, providers may choose nonselective alpha-blockade for patients with very high levels of catecholamine secretion due to irreversible receptor binding; catecholamine profile may also influence blocking strategy or duration.^19,20^ Further, we were unable to parse outcomes from patients who received combination therapy with both selective and nonselective alpha-blockade, which narrowed our analytic cohort. However, using a binary distinction allowed us to make clear comparisons between alpha-blockade strategies and strengthened our ability to control for confounding. The generalizability of our study is limited by the large percentage of patients excluded in our final analysis due to stringent inclusion criteria. In addition, as with all claims data, there is the potential for miscoding including for the diagnosis of pheochromocytoma, as granular pathology data were not available to exclude a false positive test.

Although the difference in cumulative cost between alpha-blockade strategies is insignificant, the distribution of the medication cost is not. Nonselective alpha-blockade places a greater financial burden on patients, as medication costs are frequently out-of-pocket patient expenses; the costs in this analysis did not discriminate between patient and insurance payments or co-pays, and therefore further investigation would be warranted. From a patient-centered perspective, the greater than 50-fold difference in alpha-blockade cost cannot be overlooked, especially in settings where out-of-pocket costs can impact access. Therefore, selective alpha-blockade is a more cost-effective preoperative regimen.

## Supplementary Information

Below is the link to the electronic supplementary material.

Supplementary file1 (DOCX 15 KB)
