# Supplementary material for: What is the Cost of Selectivity? Selective and Nonselective Alpha Blockade Costs Associated with Adrenalectomy for Pheochromocytoma
Source: Ann Surg Oncol. 2026 Feb 20;33(6):5651–8. doi: 10.1245/s10434-026-19255-3 (PMC13179249; doi:10.1245/s10434-026-19255-3)
Supplement: Supplementary file 1 — Supplementary file1 (DOCX 15 KB) [file 10434_2026_19255_MOESM1_ESM.docx]

**Supplementary Table 1.** Adrenalectomy approach codes

| **Open** | **Minimally invasive** | **Unknown** |
| --- | --- | --- |
| 0GB20ZX, 0GB20ZZ,  0GB30ZX, 0GB30ZZ,  0GB40ZX, 0GB40ZZ,  0GT20ZZ, 0GT30ZZ,  0GT40ZZ,  60540, 60545 | 0GB23ZX, 0GB23ZZ,  0GB24ZX, 0GB24ZZ,  0GB33ZX, 0GB33ZZ,  0GB34ZX, 0GB34ZZ,  0GB43ZX, 0GB43ZZ,  0GB44ZX, 0GB44ZZ,  0GT24ZZ, 0GT34ZZ,  0GT44ZZ,  60650 | **ICD-9 procedure codes:**   \| 0700 \| \| --- \| \| 0701 \| \| 0702 \| \| 0721 \| \| 0722 \| \| 0729 \| \| 073 \| |
